# Incidental Cervical Carcinoma Causing Obstructed Preterm Labor at 23 Weeks: A Case Report

**DOI:** 10.7759/cureus.105701

**Published:** 2026-03-23

**Authors:** Nada Douraidi, Soukaina Mouiman, Meryem Abbouch, Najia Zeraidi, Aziz Baidada

**Affiliations:** 1 Gynecology-Obstetrics and Endoscopy, Maternity Souissi, University Hospital Center Ibn Sina of Rabat, Rabat, MAR

**Keywords:** cervical cancer, chorioamnionitis, maternal sepsis, obstructed labor, preterm birth

## Abstract

Cervical cancer may remain undiagnosed during pregnancy in the absence of antenatal care, with potentially catastrophic obstetric consequences. We report an exceptionally rare case of an undiagnosed 5 cm cervical carcinoma discovered at 23 weeks of gestation, simultaneously obstructing the birth canal and complicated by chorioamnionitis-induced maternal sepsis.

A 35-year-old multiparous woman (gravida 5 para 4) with a one-year history of uninvestigated post-coital metrorrhagia and no antenatal care presented at 23 weeks and 2 days with fever, pelvic pain, and watery vaginal discharge. Examination revealed sepsis with hemodynamic instability and a 5 cm indurated cervical mass with purulent leukorrhea; transvaginal ultrasound confirmed birth canal obstruction by the mass. An emergency cesarean section was performed, and a male neonate of 400 grams was delivered, dying two hours after birth from extreme prematurity. Cervical biopsies confirmed grade 2 squamous cell carcinoma (SCC); the patient was staged as International Federation of Gynecology and Obstetrics (FIGO) IIB and scheduled for radical hysterectomy and adjuvant chemoradiation.

A cervical tumor can simultaneously obstruct labor and act as a nidus for ascending intrauterine infection, a dual mechanism not previously described. This case underscores the need for routine antenatal cervical examination and the urgent establishment of a national cervical cancer screening program aligned with international recommendations.

## Introduction

Cervical cancer is the fourth most common malignancy in women worldwide, with approximately 660,000 new cases annually [1], and represents the most common gynecological malignancy diagnosed during pregnancy, with an incidence of 0.44 to 5.08 cases per 100,000 pregnancies [2]. The diagnosis is frequently delayed, as symptoms such as vaginal bleeding or discharge are often attributed to normal gestational changes, and pelvic examination may be omitted or deferred during antenatal care [3]. When undetected, an advanced cervical carcinoma may remain clinically silent until a precipitating obstetric emergency reveals it.

Labor dystocia due to a pelvic mass is a recognized though uncommon cause of obstructed delivery. The etiology may be benign uterine leiomyomas, which are the most frequently reported, associated with significantly higher rates of dysfunctional labor and cesarean delivery [4, 5], or malignant. Cervical carcinoma and other pelvic malignancies have been described as rare causes of obstructed labor [6, 7, 8], with only a handful of cases published since Jones first reported obstructed labor due to cervical carcinoma in 1963 [7]. van Hamont and Zusterzeel described two cases where labor dystocia was the first presentation of a pelvic malignancy [6], and Ghosh and Tempe reported a retroperitoneal adenocarcinoma presenting similarly in an unbooked multipara [8], both emphasizing the critical role of routine antenatal care. Additionally, necrotic cervical tumors may provide a direct pathway for ascending infection; chorioamnionitis carries significant maternal morbidity, including sepsis and adult respiratory distress syndrome, and worsens neonatal outcomes [9].

We present a uniquely complex case of a 35-year-old multiparous woman with no antenatal care who presented at 23 weeks of gestation with obstructed preterm labor caused by an undiagnosed 5 cm cervical carcinoma, compounded by chorioamnionitis and maternal sepsis. To our knowledge, this combination has not previously been described in the literature.

## Case presentation

We report the case of a 35-year-old woman with no significant past medical history who had been married for 19 years. Her obstetric history was notable for gravida 5, para 4, and four prior uncomplicated vaginal deliveries, all resulting in live births. She reported a one-year history of post-coital metrorrhagia, for which she had not sought medical attention. Her current pregnancy, at 23 weeks and 2 days of gestation, was unmonitored.

The patient presented to our obstetric emergency department with a three-day history of fever, pelvic pain, and watery vaginal discharge, for which she had not previously consulted a physician. On clinical examination, she exhibited signs consistent with sepsis, including a fever (temperature of 39.2°C), tachycardia (heart rate of 118 beats per minute), tachypnea (respiratory rate of 22 breaths per minute), and hypotension (blood pressure of 88/56 mmHg). She was diaphoretic and appeared acutely unwell. Uterine tenderness was noted on abdominal palpation.

**Figure 1:**
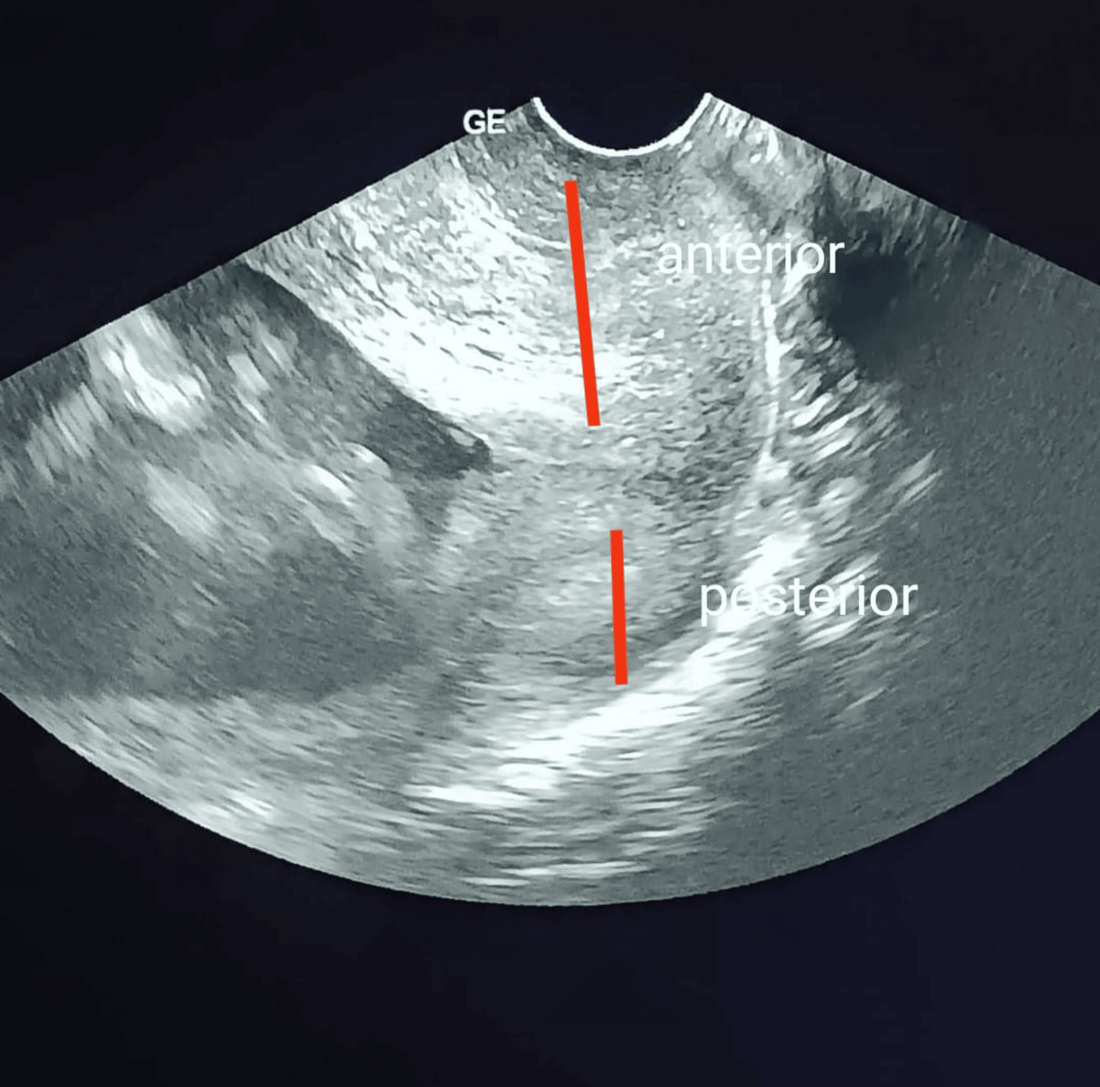
Transvaginal ultrasound showing an enlarged heterogeneous cervix measuring 5 cm, involving both anterior and posterior lips, causing mechanical obstruction of the birth canal.

**Figure 2:**
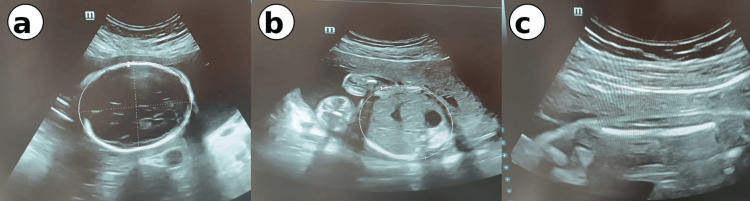
Fetal biometry ultrasound at 23 weeks of gestation (a) Head circumference = 21.15 cm at 22w5d; (b) Abdominal circumference = 17.40 cm, 22w3d; (c) Femur length = 3.43 cm, 20w5d; Estimated fetal weight = 454g w: weeks; d: days

Gynecological examination revealed a markedly indurated cervical mass measuring approximately 5 cm, involving both the anterior and posterior lips of the cervix. The external cervical os was dilated to 1 cm. Foul-smelling, greenish cervical discharge was noted, consistent with purulent leukorrhea. Transvaginal ultrasound confirmed an enlarged, heterogeneous cervix measuring 5 cm in its greatest diameter, with the mass involving both cervical lips and causing mechanical obstruction of the birth canal, consistent with obstructed preterm labor (Figure 1). Pelvic ultrasound further demonstrated a singleton live intrauterine pregnancy with a fetal weight estimated at 454 grams, corresponding to the stated gestational age (Figure 2).

A diagnosis of intrauterine infection (clinical chorioamnionitis) complicated by maternal sepsis was established on the basis of the clinical findings. In view of the obstructed labor caused by the cervical mass, vaginal delivery was deemed impossible. Following thorough counseling of the patient and her family regarding the severe maternal and fetal prognosis, including the periviable gestational age and the high risk of fetal non-survival, informed consent was obtained for emergency cesarean delivery.

**Figure 3:**
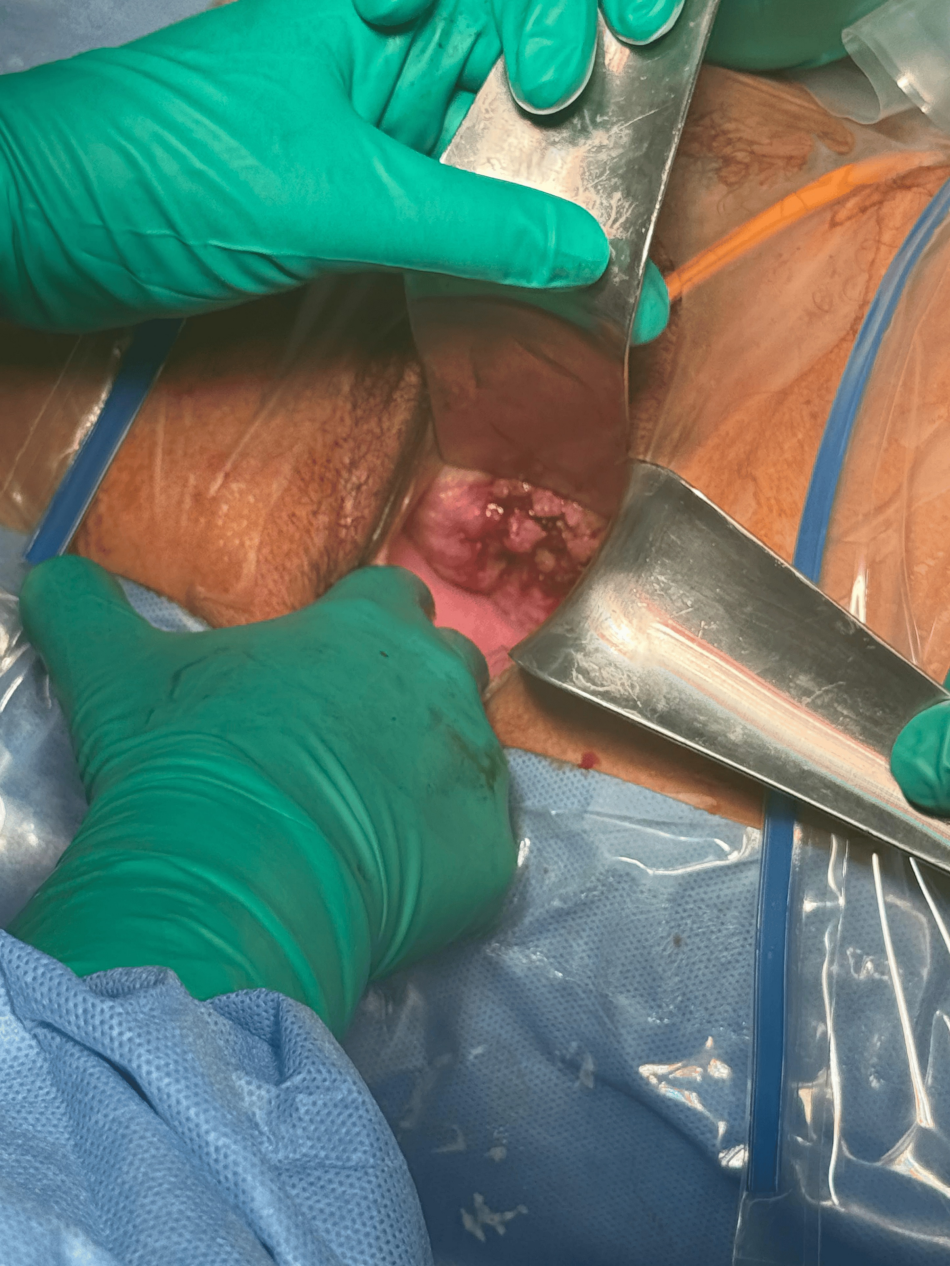
Examination under anesthesia following cesarean delivery demonstrating the exophytic cervical mass.

**Figure 4:**
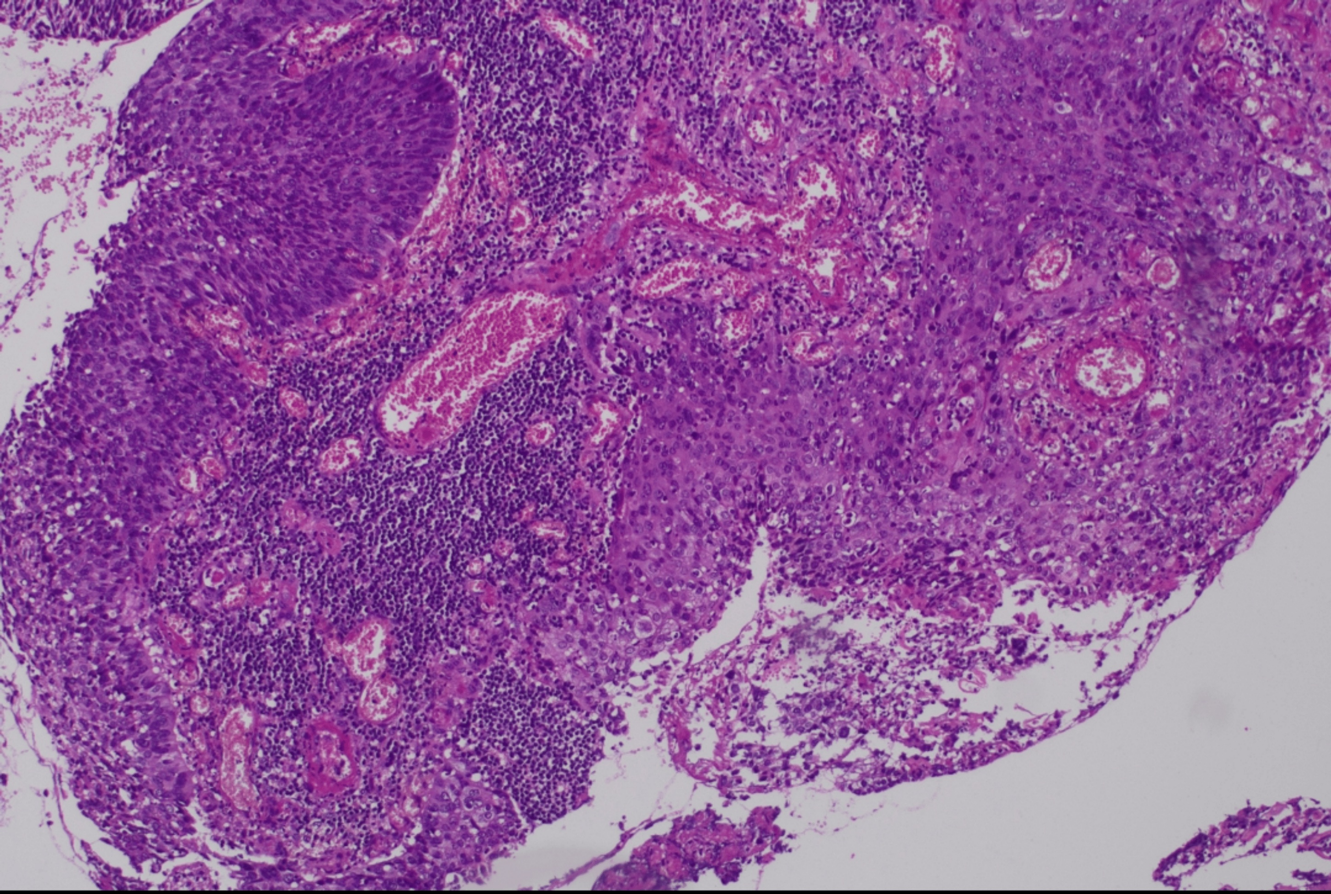
Histopathological examination of the cervical biopsy (hematoxylin and eosin stain, original magnification ×100)

An emergency cesarean section was performed under general anesthesia, with simultaneous initiation of broad-spectrum intravenous antibiotic therapy. A male fetus weighing 400 grams was delivered. Despite full resuscitative efforts, the neonate died two hours after birth, attributable to extreme prematurity at 23 weeks of gestation. Intraoperatively, the amniotic fluid was turbid and foul-smelling, consistent with established intrauterine infection. Following delivery, a speculum examination was performed under anesthesia to allow direct and complete visualization of the cervical mass (Figure 3). Punch biopsies of the cervical lesion were obtained and submitted for histopathological analysis, which subsequently confirmed a diagnosis of grade 2 squamous cell carcinoma (SCC) of the uterine cervix (Figure 4).

Following histological confirmation, the patient was referred to the gynecologic oncology department for staging and oncological management. Clinical staging according to the 2018 International Federation of Gynecology and Obstetrics (FIGO) classification was performed, and the tumor was staged as FIGO Stage IIB, based on parametrial involvement identified on clinical examination [10]. Contrast-enhanced computed tomography of the chest, abdomen, and pelvis revealed no pathological lymphadenopathy and no evidence of distant metastasis. However, reliable assessment of the uterine cervix on imaging was limited due to the short postpartum interval and post-surgical changes. Following multidisciplinary tumor board discussion, the patient was scheduled to undergo radical hysterectomy (Wertheim-Meigs), bilateral salpingo-oophorectomy, and bilateral pelvic lymphadenectomy, with adjuvant concurrent chemoradiation to be considered based on final surgical pathology.

## Discussion

This case illustrates a rare and extreme clinical scenario in which an advanced, previously undiagnosed cervical carcinoma simultaneously presented as a mechanical obstruction to preterm labor and as the portal of entry for a life-threatening intrauterine infection. Each of these components alone constitutes a serious obstetric emergency; their co-occurrence at a periviable gestational age of 23 weeks makes this case exceptionally rare and clinically instructive.

Obstructive labor secondary to a pelvic or cervical mass is an infrequently reported condition. van Hamont and Zusterzeel described two cases in which labor dystocia was the first presentation of an underlying pelvic malignancy, including one case of grade 2 SCC of the cervix causing failure of cervical dilation despite adequate uterine contractions [6]. Similarly, Ghosh and Tempe reported a case of retroperitoneal adenocarcinoma of unknown origin presenting as obstructed labor at term in an unbooked multipara, with the diagnosis established only after emergency cesarean section [8]. In both cases, as in ours, the malignancy had gone entirely undetected due to the absence of routine antenatal surveillance. Benign pelvic masses, particularly uterine fibroids, are also well-documented causes of dysfunctional labor and increased cesarean delivery rates [4, 5] and must remain in the differential diagnosis when labor obstruction is encountered.

What critically distinguishes our case from all previously reported cases of malignancy-related labor obstruction is the gestational age at presentation. At 23 weeks and 2 days, this patient was at the threshold of periviability. No comparable case in the literature describes obstructed labor from a cervical malignancy at such an early gestational age. Previously reported cases of cervical cancer discovered at the time of labor involve term or near-term pregnancies [6, 7], affording the neonate a substantially better prognosis. The extreme prematurity of the neonate in our case, combined with the severity of the concurrent infection, resulted in neonatal death within two hours of delivery despite full resuscitative efforts.

A second and equally critical feature of this case is the presence of clinical chorioamnionitis complicated by frank maternal sepsis. In patients with bulky cervical carcinoma, the tumor surface is frequently ulcerated, necrotic, and heavily colonized with polymicrobial flora from the lower genital tract. This creates a direct anatomical pathway for ascending infection into the intrauterine compartment, independent of membrane status. In our patient, the necrotic 5 cm cervical mass almost certainly acted as the primary nidus for intrauterine infection, facilitating bacterial ascent and ultimately triggering maternal sepsis. This pathophysiological mechanism, tumor-mediated ascending chorioamnionitis, is rarely discussed in the literature on cervical cancer in pregnancy and represents a novel and clinically important observation. Intra-amniotic infection is well recognized to carry significant maternal morbidity, including endometritis, peritonitis, sepsis, and adult respiratory distress syndrome, underscoring the urgency of prompt management [9].

The management of this case required simultaneously addressing two life-threatening conditions: obstructed labor and septic shock. As the cervical tumor rendered vaginal delivery mechanically impossible, an emergency cesarean section was the only viable option. This approach is concordant with established guidelines recommending cesarean delivery for all patients with unresected cervical cancer, both to avoid birth canal obstruction and to prevent tumor cell seeding at episiotomy sites, a phenomenon associated with significantly higher rates of locoregional recurrence [11].

The absence of any antenatal care in this patient is a pivotal factor in the sequence of events that led to this emergency. The patient had experienced post-coital metrorrhagia for one year prior to her presentation, a symptom highly characteristic of invasive cervical carcinoma, without ever seeking medical evaluation. Had routine antenatal care been initiated, a pelvic examination would very likely have identified the cervical mass at an earlier gestational age, enabling planned oncological management, preservation of fetal viability, and avoidance of the septic complication. As both van Hamont et al. [6] and Ghosh and Tempe [8] independently concluded, adequate and repeated pelvic examination throughout routine antenatal care is essential to detect pelvic masses early, reduce obstructed deliveries, and prevent late and potentially fatal complications of undiagnosed malignancy.

## Conclusions

We report an exceptionally rare and life-threatening case of undiagnosed bulky cervical carcinoma presenting as obstructed preterm labor at 23 weeks, compounded by chorioamnionitis-induced maternal sepsis, a combination not previously described in the literature. This case underscores that pelvic malignancy must always be considered in the differential diagnosis of unexplained labor dystocia and that post-coital bleeding must never go uninvestigated in pregnancy. An emergency cesarean section was lifesaving for the mother while enabling a histological diagnosis of the cervical mass. Most critically, this case highlights the urgent need for a national cervical cancer screening program that adheres to international recommendations, including systematic human papillomavirus testing and cervical cytology for all women of reproductive age as the only sustainable strategy to prevent such tragedies and reduce the disproportionate burden of late-stage cervical cancer in our population.
